# Predictors of stakeholders’ intention to adopt nutrigenomics

**DOI:** 10.1186/s12263-020-00676-y

**Published:** 2020-09-22

**Authors:** Muhammad Adzran Che Mustapa, Latifah Amin, Lynn J. Frewer

**Affiliations:** 1 Pusat Citra Universiti, Universiti Kebangsaan Malaysia, 43600 UKM Bangi, Selangor Malaysia; 2 The Institute of Islam Hadhari, Universiti Kebangsaan Malaysia, 43600 UKM Bangi, Selangor Malaysia; 3 School of Natural and Environmental Sciences, Newcastle University, Newcastle upon Tyne, UK

**Keywords:** Predictors, Stakeholders, Intention, Nutrigenomics, Malaysia

## Abstract

**Background:**

Nutrigenomics is an emerging science that studies the relationship between genes, diet and nutrients that can help prevent chronic disease. The development of this science depends on whether the public accept its application; therefore, predicting their intention to adopt it is important for its successful implementation.

**Objective:**

This study aims to analyse Malaysian stakeholders’ intentions to adopt nutrigenomics, and determines the factors that influence their intentions.

**Methods:**

A survey was conducted based on the responses of 421 adults (aged 18 years and older) and comprising two stakeholder groups: healthcare providers (*n* = 221) and patients (*n* = 200) who were located in the Klang Valley, Malaysia. The SPSS software was used to analyse the descriptive statistics of intention to adopt nutrigenomics and the SmartPLS software was used to determine the predicting factors affecting their decisions to adopt nutrigenomics.

**Results:**

The results show that the stakeholders perceived the benefits of nutrigenomics as outweighing its risks, suggesting that the perceived benefits represent the most important direct predictor of the intention to adopt nutrigenomics. The perceived risks of nutrigenomics, trust in key players, engagement with medical genetics and religiosity also predict the intention to adopt nutrigenomics. Additionally, the perceived benefits of nutrigenomics served as a mediator for four factors: perceived risks of nutrigenomics, engagement with medical genetics, trust in key players and religiosity, whilst the perceived risks were a mediator for engagement with medical genetics.

**Conclusion:**

The findings of this study suggest that the intentions of Malaysian stakeholders to adopt nutrigenomics are a complex decision-making process where all the previously mentioned factors interact. Although the results showed that the stakeholders in Malaysia were highly positive towards nutrigenomics, they were also cautious about adopting it.

## Introduction

Scientists are examining the impact of nutrition on maintaining health and preventing chronic disease [1]. Traditionally, nutrition research has been concerned with the provision of nutrients to sustain population health. More latterly, nutrition research can be applied to improve peoples’ health through individualisation of diets. Thus, research into nutrition is increasingly concerned with health promotion, disease prevention, and improving mental and physical performance [2]. As people respond differently to diets depending on their lifestyle, environment and genetic makeup, personalised nutrition involves adapting dietary intake to suit their individual needs [3]. Several aspects of personalised nutrition have been successfully implemented in the field of nutrition, such as advice based on dietary intake, lifestyle, phenotype and personal goals [4–6].

More recently, it has been established that an individual’s genetic background can affect nutrition related or dependent disorders [7]. Subsequently, diet has been reported as having the ability to affect metabolic processes at the molecular level. These findings have led to the evolution of new terminologies: nutrigenetics and nutrigenomics [8]. Marcum [9] proposed that nutrigenetics and nutrigenomics have complementary roles. The term nutrigenetics was first presented by Brennan in 1975 [10]. Nutrigenetics is a facet of personalised nutrition that examines the impact of genetic variations, notably related to the single-nucleotide polymorphism (SNP), on people’s response to dietary intake [9]. As molecular techniques have advanced, nutrigenetics has led to nutrigenomics. The latter is a scientific approach that integrates nutritional sciences with genomics and includes the application of other ‘omics’ technologies (metabolomics, proteomics and transcriptomics) [11]. Nutrigenomics represents the study of the effect of foods and food constituents on gene expression and health [12, 13]. The technology can determine the effect of nutrients on protein synthesis, specifically DNA transcription and translation processes. This provides insights into how nutrients can affect the expression of genes involved in the regulation of important metabolic pathways, which influence people’s health [14]*.* An understanding of the gene-nutrient interactions may help in the prevention of disease [15–17], assuming that diets can be developed which align with the nutritional requirements of the individual. This can improve the effectiveness of personalised and targeted approaches with respect to dietary health promotion [18], with successful outcomes for some non-communicable diseases such as cardiovascular disease [19].

Although there are more challenges to implementing personalised nutrition based on genetic make-up compared to those developed from phenotype or personal goals, there has been a growing interest in the use of individual-DNA information to tailor lifestyle interventions in the last few decades [20, 21]. Increased understanding of the gene-nutrition relationship, which offers opportunities for health promotion and disease prevention, has dramatically boosted nutrigenomics research [22–25]. A study in the USA and in the European countries has reported that the public are generally interested in genetic testing and personal genomics [26–28], as well as in adopting nutrigenomics-based personalised nutrition [27, 29, 30]. In a Canadian study by Castle et al. [31] and Marcotte et al. [32], most members of the public surveyed had a favourable interest in nutrigenomics, and perceived potential health benefits to be associated with nutrigenetic testing. Another study indicated that individuals perceived DNA-based dietary advice to be more valuable and understandable than food-based dietary guidelines, which motivated them to change their diet using gene-based personalised nutrition information [20, 33]. Moreover, genetic testing services are currently widely available and not only limited to developed countries. In Malaysia, a total of twenty genetic testing laboratories, both public and private, offer a variety of genomic services (including genetic testing and counselling) [34].

The relationship between technological innovations and societal responses has a long and complex history, and there are various factors influencing public perception and adoption of new technologies [35–39]. Consumers do not always understand the added value of new and complex products, so a structured evaluation of their perspectives is required [40]. Predicting people’s intentions regarding the adoption of new food technologies is important and could determine the development of the technology, its subsequent successful implementation and commercialisation [41]. Public support for a new enabling technology and its application is an important and necessary condition for its successful application in society. Pin [35] contends that if genomics technology can be used to predict disease and prescribe preventive diets based on a person’s genetic profile, it is, ‘a priori’, important to study people’s intentions to adopt such personalised diets and modify applications in accordance with consumers’ expectations. The social context surrounding a technology is, therefore, likely to be one of the most important determinants of its future development and application.

Previous researchers have explored a range of factors influencing public perceptions of food innovations [37, 40]. Some studies have attempted to combine various psychological determinants into a predictive model of behavioural intentions [37, 42]. The literature on public perceptions of genomics focuses on predictors such as perceptions of cost and benefit, the positive and negative effects of a specific technology and attitudes towards that technology [35]. It is reported that when an individual perceives there to be a benefit from nutrigenomics-based personalised nutrition, there is an increase in the positive affect and in their belief that the technique can have desirable consequences [35]. This, in turn, strengthens the individual’s conviction to adopt nutrigenomics-based personalised nutrition. This paper concentrates on the factors influencing Malaysian stakeholders’ intention to adopt nutrigenomics. To our knowledge, research into the determinants of the intention to adopt nutrigenomics has not previously been conducted in Malaysia.

## Theoretical framework

The theoretical framework in this study is a modified version of the model proposed by Chen and Li [43], who generated theirs based on Bredahl [36] and Fishbein’s multi-attribute attitude model [44]. Attitude is comprised of the affective, cognitive and behavioural components [44, 45]. Pennington [46] asserted that the affective and cognitive components are framed to reflect the positive or negative evaluations of an entity or product according to people’s beliefs. The cognitive and affective components in Chen and Li’s [43] framework are perceived benefits and perceived risks that have been reported in attitude models towards nutrigenomics [40] and attitude models towards other contemporary issues such as GM foods [47, 48].

According to Shi and Kim [49], the theory of planned behaviour (TPB) has overlapping concepts with the risk perception attitude (RPA) framework, where perceived risk was reported as a predictor for behavioural intention. Slovic et al. [50] and Loewenstein [51] stressed that perceived risks should be conceptualised as the complementary process of both affective (‘feelings’) and cognitive (‘analytical’) components when making judgements about potential hazards. The affective component is translated into perceived consequences of the hazard should it occur (cognitive appraisals) [50]. Both affective and cognitive judgements have been shown to predict behavioural intentions [50]. In the research presented here, perceived benefit is also conceptualised as representing both the affective [50] and cognitive dimensions [52].

It should be noted that the variable subjective norms in Fishbein’s multi-attribute attitude model [44] were not included in this study’s framework, as this variable was found to be inconsistent in predicting attitude and intention [36, 48], which was subsequently not incorporated in models related to attitude and intention to adopt nutrigenomics [35] and other studies on gene technology [53, 54]. This is because gene technology is considered a complex issue not commonly discussed in immediate circles such as family members [55]. The same goes for the perceived control factor, which was also not a consistent predictor for attitude to contemporary technologies [36] and was not adopted in other studies related to gene technology [35, 43, 47, 56–58]. Even though Fishbein’s multi-attribute attitude model [44] is useful in understanding attitude and intention, many other researchers have incorporated other factors that are also important in explaining attitude to provide better variance [43, 48]. Attitudes and intention to use new innovations have been shown to be influenced by more general attitudes and values [40, 43, 59–61].

**Fig. 1:**
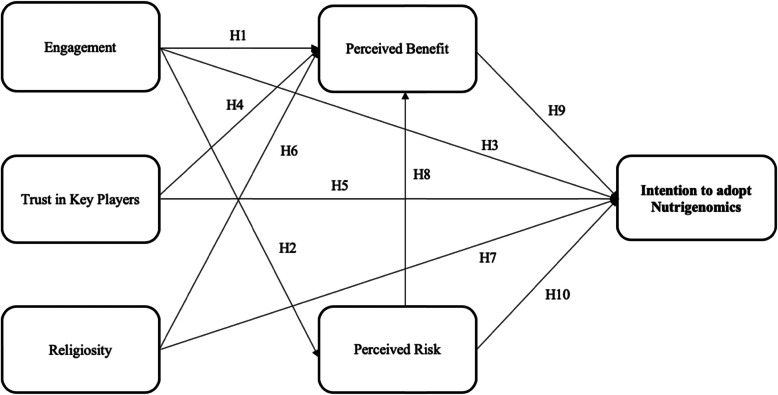
The conceptual framework for stakeholders’ intention to adopt nutrigenomics. H1-H10 refers to the related hypotheses

The conceptual framework for this study is presented in Fig. 1. It comprises of potential causes that are known to affect behavioural intention [43]. Perceptions of the risks and benefits of technologies in healthcare are considered to be the predominant factors contributing to their successful adoption. When individuals or targeted groups are more inclined to perceive the positive attributes, they are more likely to embrace the technology [62]. If individuals perceive there are potential benefits from a behaviour or choice, the risk associated with this behaviour or choice is perceived as lower [63]. A study by Berezowska et al. [25] in eight European countries (Greece, Spain, the Netherlands, Ireland, the UK, Germany, Poland and Norway) confirmed that consumers’ intention to adopt nutrigenomics-based personalised nutrition depends more heavily on its benefits than its risks. Consumer rejection of adopting nutrigenomics-based personalised nutrition may compromise the potential benefits of the technology [25]. The concept of risk perception is frequently linked to issues of safety and encompasses the short and long-term impacts on the environment, human health, and moral and societal issues [35, 64]. Risk and benefit perceptions are complex, often mutually dependent, and have an inverse association [64–66]. Although it would be interesting to hypothesise two-way relationships between perceived risks and benefits, partial least squares structural equation modelling (PLS-SEM) has the limitation that the paths between the latent constructs can only head in a single direction [67], resulting in the path was proposed from perceived risks to benefits. This was after taking into account the inference by Frewer, Howard and Shepherd [68] that people tend to focus more on the benefits of technology than its risks. There is limited research on the relationship between the two variables related to nutrigenomics; however, the research by Costa-Font and Gil [47] and Prati et al. [48] showed that perceived benefits mediated the influence of perceived risk on attitudes to GM food.

Trust, knowledge and general attitude were significant predictors in Chen and Li’s model [43]. In this study, the trust factor was included, as Siegrist [53, 69] suggested that it has an effect on the perceived benefits and risks of gene technology. Poínhos et al. [70] reported that the trust factor represents the strongest significant predictor for public intention to adopt nutrigenomics in nine European countries (Germany, Greece, Ireland, Poland, Portugal, Spain, the Netherlands, the UK, and Norway). Moreover, Berezowska et al. [25] highlighted the distinct role of trust in the decision-making process with regard to consumer adoption of personalised nutrition services based on nutrigenomics in eight European countries (Greece, Spain, the Netherlands, Ireland, the UK, Germany, Poland, and Norway). This suggests the need for the trust factor to be included in research on consumer behaviour associated with nutrigenomics. Therefore, due to its importance, trust in key players was included in this study’s framework.

The influence of knowledge on attitudes towards science and technology [71–73] and attitudes towards nutrigenomics and genetic testing [32, 35, 68–72] have been found to be inconsistent in past studies. Gaskell et al. [65] recommended the ‘engagement’ concept based on ‘issue public’ and proved that the variable was consistently associated with higher individual support for six applications of biotechnology. Other researchers have used a similar concept, which they called the ‘attentive public’, in their survey [74, 75]. Pin [35] reported a strong association between involvement and positive affect and benefit ratios of personalised nutrition based on nutrigenomics. So, the knowledge variable in Chen and Li’s model [43] was replaced with engagement.

The general attitude factor in Chen and Li’s [43] model was not included in this study, as the construct consisted of one item each representing four sub-constructs: attitude to science and technology, attitude towards nature, food neophobia and attitude to health. Other studies have cited that the sub-constructs represented separate factors [36, 56, 58, 76]. The four separate variables were not included in this study’s model as the model would be too complicated; it will be considered in future studies. Instead, religiosity was added as representative of value systems. Cultural values have been reported to influence people’s attitudes and their behaviour with regard to food choice decisions and eating habits [77, 78]. For gene technology-related applications, Brody [79] highlighted the importance of including religious or cultural traditions, as people tend to judge the technology using ethical perspectives as well as the benefits and risk perceptions. Religion is part of the cultural elements and has a considerable influence on people’s values, habits, attitudes and lifestyles, which affects their decision-making behaviour [40, 80–82]. The majority of Malaysians are Malay, who are also Muslims (63.1%) [83]. The Islamic religion is an ongoing part of the daily life of Muslims and is embedded in their cultural and personal values [78]. Malaysians have acknowledged that they are highly religious, and they refer to religion during most of their day-to-day decision-making processes [58]. Regardless of their specific faith, Malaysians describe themselves as highly religious [56]. Previous findings showed that people who are more religious tend to be more judgemental with regard to biotechnology issues [56, 84]. Previous studies suggested that religiosity influences perceptions of the benefits and risks of technologies, and attitudes to information-seeking behaviour [85]. Only by recognising the validity of these concerns, can technology be accepted by society [58, 86]. Moreover, there has been no specific study on the influence of religiosity on attitude and intention to adopt nutrigenomics. Thus, it is crucial that the role of religiosity be included in the framework. This will contribute to the body of knowledge on its possible influence. The relationships between all the variables were hypothesised as follows based on the significant correlation between the variables using Pearson correlation analyses (Table 1).

**Table 1:** The correlation matrix amongst factors

	Engagement in medical genetics	Trust in key players	Religiosity	Perceived benefit of nutrigenomics	Perceived risk of nutrigenomics	Intention to adopt nutrigenomics
Engagement with medical genetics	1					
Trust in key players	0.206**	1				
Religiosity	0.146**	0.249**	1			
Perceived benefit of nutrigenomics	0.289**	0.422**	0.237**	1		
Perceived risk of nutrigenomics	−0.127**	−0.059	−0.050	−0.150**	1	
Intention to adopt nutrigenomics	0.342**	0.435**	0.296**	0.609**	−0.212**	1

*P < 0.05; **P < 0.01

H1: When stakeholders have better engagement with medical genetics, they will perceive more benefits associated with nutrigenomics.

H2: When stakeholders have better engagement with medical genetics, they will perceive fewer risks associated with nutrigenomics.

H3: When stakeholders have better engagement with medical genetics, they will have higher intentions to adopt nutrigenomics.

H4: When stakeholders have more trust in key players involved in medical genetics, they will perceive more benefits associated with nutrigenomics.

H5: When stakeholders have more trust in key players involved in medical genetics, they will have higher intentions to adopt nutrigenomics.

H6: When stakeholders view themselves as having a higher level of religiosity, they will perceive more benefits associated with nutrigenomics.

H7: When stakeholders view themselves as having a higher level of religiosity, they will have greater intentions to adopting nutrigenomics.

H8: When stakeholders perceive higher risks associated with nutrigenomics, they will perceive fewer benefits to adopting nutrigenomics.

H9: When stakeholders perceive higher benefits associated with nutrigenomics, they will perceive greater intentions to adopt nutrigenomics.

H10: When stakeholders perceive higher risks associated with nutrigenomics, they will perceive lower intentions to adopt nutrigenomics.

## Methods

### Data collection

Data was collected via face-to-face surveys held with 421 adult respondents (aged 18 years and over) from June to September 2017 in major public hospitals in the Klang Valley, Malaysia. This region was selected as the study area because it is a main centre of socio-economic development in Malaysia and has residents from a diverse range of demographic backgrounds. Two different groups of people directly involved with medical genetics—healthcare providers (*n* = 221) and patients (*n* = 200)—were invited to participate and respondents were selected using the stratified random sampling method. The healthcare providers comprised medical practitioners, geneticists, registered dietitians/nutritionists, pharmacists, nurses and medical laboratory technicians, whilst the patients were individuals who received treatment at the hospital, or family members who represented them.

The questionnaires were handed out personally to respondents by three genetics graduate enumerators who were trained to be neutral and un-biassed on their stance towards nutrigenomics. Prior to completing the questionnaires, the respondents were given a brief un-biassed introduction on nutrigenomics, its application and possible issues and limitations (Additional file 1). This approach was suggested by Kelley [87] to assess unsophisticated public attitudes on complex issues such as modern biotechnology. Sturgis et al. [88] have shown that the provision of information prior to the survey does not affect people’s attitudes to biotechnology. Using this approach, the respondents do not have to know anything about medical genetic concepts and developments in the past. They were introduced to the basic concepts and examples of medical genetics applications. This approach is appropriate for sophisticated respondents as well as unsophisticated respondents, and allows the researchers to use sophisticated statistical multivariate. By using a multiplicity of questions, measurement errors are reduced [87]. It should be noted that there is a possibility that some respondents might have asked more questions and, therefore, received more information. This limitation was minimised by only recruiting three well-trained enumerators. Even if some respondents might have received more information, as long as it was given in un-biassed form, it should not have made significant differences to their attitudes [89].

### The survey instrument

The multi-dimensional instrument consisting of 48 items was adapted from earlier research [53, 56, 57, 90] with some refinements to make the items more relevant for nutrigenomics [Additional file 2]. The instrument incorporated six variables: engagement with medical genetics [56], trust in key players [53, 56], religiosity [56], perceived benefits, perceived risks [53, 56, 57, 90] and behavioural intention [56].

In this study, the engagement with medical genetics (CR = 0.823) is defined as using a combination of awareness, knowledge and past and intended information-seeking behaviour (Gaskell et al. [91]). For the knowledge sub-construct, respondents were asked whether the ten statements regarding the concepts and facts about medical genetics were true or false. As for awareness, respondents were asked whether they had heard of five applications of medical genetics and two related developments in Malaysia (adapted from [91]) Three items which referred to the past and intended information-seeking behaviour were included, [91]; with each item being measured on a 7-point scale, ranging from 1 (strongly disagree) to 7 (strongly agree). Religiosity (CR = 0.917) comprised four items involving the importance of religion [92] and religious rites [93] in the respondents’ life. Trust in key players (CR = 0.868) was assessed by asking the respondents three items on the extent to which service providers, i.e., government departments involved in medical genetics regulation, and companies, were perceived to have done a good job for society [91].

The remaining constructs: perceived benefits, perceived risks and intention to adopt were specific to nutrigenomics. The perceived benefits of nutrigenomics scale (CR = 0.823) consisted of six items. Each item was measured on a 7-point scale, ranging from 1 (strongly disagree) to 7 (strongly agree). The measure for perceived risk of nutrigenomics (CR = 0.896) was obtained by using five items and each item was measured on a 7-point scale, ranging from 1 (strongly disagree) to 7 (strongly agree). Intention to adopt nutrigenomics (CR = 0.917) was measured by five items with each item measured on a 7-point scale, ranging from 1 (strongly disagree) to 7 (strongly agree). A brief introduction regarding nutrigenomics and its applications (such as nutrigenomics-based personalised nutrition and the purpose of nutrigenomics) was presented to the respondents prior to administering the questionnaire. In addition, the respondents were permitted to ask questions so that they were fully informed about the study and understood the benefits and risks related to the application of nutrigenomics.

### Statistical analysis

The data was analysed using the SmartPLS software (version 3.2.7) to evaluate the significant predictors and assess the relationships between the constructs. Four steps were followed: testing the validity and reliability of the constructs, discriminant validity analysis, analysing the structural relationships and assessing the fitness of the overall model. PLS-SEM is a well-established technique that has been frequently applied by many researchers across a variety of disciplines [67, 94–97]. PLS-SEM has been chosen in this study, as it is rated as being more suitable for predictive analysis, and can maintain the relevant indicator variables without compromising the predictive accuracy and robustness of *R*^2^ [98]. Additionally, it has been shown as being more rigorous with fewer issues in identification, as well as being able to curtail the problem of residual variances related to endogenous constructs [67]. Covariance based SEM (CB-SEM) is limited because of the elimination of relevant indicator variables in relation to increased model fit at the expense of the construct validity [98].

## Results

### Descriptive analysis

The Malaysian stakeholders claimed they were moderately engaged with modern biotechnology activities (mean score 5.05), and that they trusted the key actors: service providers, industries and government regulators (mean score 5.31) (Table 2). They also rated themselves as highly religious, regardless of the religion followed (mean score 6.31). Regarding the application of nutrigenomics, they perceived it as highly beneficial (mean score 5.50) with a moderate risk (mean score 3.44), which was translated into a high intention to adopt nutrigenomics (mean score 5.41).

**Table 2:** Mean scores for intention to adopt nutrigenomics and its predictors

Dimension	Mean (standard deviation)	Interpretation
Engagement with medical genetics	5.05 (1.49)	*Moderate
Trust in key players	5.31 (0.94)	High
Religiosity	6.31 (0.97)	High
Perceived benefits of nutrigenomics	5.50 (1.08)	High
Perceived risks of nutrigenomics	3.44 (1.21)	Moderate
Intention to adopt nutrigenomics	5.41 (1.16)	High

1-2.99: Low; 3.00-5.00: Moderate; 5.01-7.00: High

*0-3.33: Low, 3.34-6.66: Moderate, 6.67-10: High

### Measurement model

The fundamental first step in developing the measurement model was to assess the convergent validity though the following criteria: the factor loadings, composite reliability and average variance extracted (AVE) [97, 99]. As shown in Table 3, the standardised loading of all items was greater than 0.6 for each factor, and, as recommended by Chin et al. [100], items with a loading value of 0.7 and above were considered significant. The composite reliability (CR) values of all factors exceeded the recommended value of 0.7. Similarly, AVE scores above 0.5 and is considered acceptable [67, 97].

**Table 3:** Reliability and validity of constructs

Factor and item	Standardised factor loading	Composite reliability (CR)	Average variance extracted (AVE)
Engagement with medical genetics			
1. Awareness	0.83	0.823	0.608
2. Knowledge	0.75		
3. Past and intended information-seeking behaviour	0.68		
Trust in key players			
1. Healthcare providers have done a good job for society	0.75	0.868	0.689
2. Government has done a good job for society	0.87		
3. Companies have done a good job for society	0.71		
Religiosity			
1. Religion is important in my life	0.87	0.917	0.734
2. Religious views are important to make decisions about controversial issues	0.83		
3. Praying is important in my life	0.78		
4. Reading scriptures is important in my life	0.86		
Perceived benefit of nutrigenomics			
1. Make people healthier	0.84	0.936	0.710
2. Bring cure for chronic diseases	0.82		
3. Enhance the quality of life	0.86		
4. Benefits to future generations	0.78		
5. Benefits exceed the risks	0.78		
6. Solve problems that cannot be solved by conventional methods	0.71		
Perceived risk of nutrigenomics			
1. Worry about unknown consequences	0.69	0.896	0.525
2. More harm than good for society	0.68		
3. Will lead to any danger to society	0.84		
4. Long-term effects	0.81		
5. Worry about safety of the application	0.72		
6. Threatening the natural order of things	0.73		
7. Gives rise to ethical issues	0.76		
8. “Playing God”	0.65		
Intention to adopt nutrigenomics			
1. Willingness to take	0.81	0.917	0.688
2. Willingness to support	0.75		
3. Willingness to recommend	0.73		
4. Willingness to pay if I can pay	0.78		
5. Willingness to take if it is cheaper	0.67		

Discriminant validity denotes the extent to which the construct is empirically different from other constructs [67, 101]. It compares the square root of the AVE of a particular construct with the correlation between that particular construct and the other constructs. From Table 4, we can see that the value of the square root of the AVE (diagonal values) of each construct is higher than its corresponding correlation coefficient, indicating adequate discriminant validity [102]. In addition, the heterotrait-monotrait ratio of correlations (HTMT) is an alternative approach to assessing discriminant validity in PLS-SEM. This method has been reported to have a superior performance compared with the Fornell-Larcker criterion [103]. For the first criterion, the HTMT value should be lower than 0.85 (indicating a stricter threshold) or 0.90 (indicating a more lenient threshold) or should be significantly smaller than 1 [104–106]. As shown in Table 5, all HTMT values were below 0.85, thus indicating good discriminant validity.

**Table 4:** Discriminant validity

	Engagement with medical genetics	Intention to adopt nutrigenomics	Perceived benefit of nutrigenomics	Perceived risk of nutrigenomics	Religiosity	Trust in key players
Engagement with medical genetics	**0.778**					
Intention to adopt nutrigenomics	0.359	**0.830**				
Perceived benefit of nutrigenomics	0.311	0.615	**0.843**			
Perceived risk of nutrigenomics	−0.140	−0.264	−0.198	**0.729**		
Religiosity	0.158	0.309	0.236	−0.078	**0.857**	
Trust in key players	0.244	0.460	0.437	−0.117	0.250	**0.831**

Values on the diagonal (in bold) are square root of the AVE whilst the off-diagonals are correlations

**Table 5:** Heterotrait-monotrait (HTMT) results

	Engagement with medical genetics	Intention to adopt nutrigenomics	Perceived benefit of nutrigenomics	Perceived risk of nutrigenomics	Religiosity	Trust in key players
Engagement with medical genetics	-					
Intention to adopt nutrigenomics	0.487	-				
Perceived benefit of nutrigenomics	0.379	0.666	-			
Perceived risk of nutrigenomics	0.189	0.223	0.177	-		
Religiosity	0.200	0.334	0.262	0.083	-	
Trust in key players	0.299	0.525	0.505	0.168	0.301	-

### Overall model fitness

In this study, the overall model fit was used to assess the validity and explanatory power of the model. This was done by assessing the standardised root mean square residual (SRMR) and normed fit index (NFI) [107]. The SRMR value displayed in Table 6 is 0.071, which is below the threshold value of 0.08 and can, therefore, be considered as a good fit and adequate for PLS path models [108]. Further, the NFI value is 0.763, which is also regarded as an acceptable fit, as it is closer to 1 [109].

**Table 6:** Structural relationships within the model

Research hypothesis	*ß*	*t* value	Conclusion
Perceived benefit of nutrigenomics ➔ Intention to adopt nutrigenomics	0.433	9.889***	Supported
Perceived risk of nutrigenomics ➔ Intention to adopt nutrigenomics	−0.126	3.541***	Supported
Trust in key players ➔ Intention to adopt nutrigenomics	0.190	4.337***	Supported
Engagement with medical genetics ➔ Intention to adopt nutrigenomics	0.140	3.766***	Supported
Religiosity ➔ Intention to adopt nutrigenomics	0.127	2.803**	Supported
Perceived risk of nutrigenomics ➔ Perceived benefit of nutrigenomics	−0.122	2.554*	Supported
Religiosity ➔ Perceived benefit of nutrigenomics	0.109	2.332*	Supported
Engagement with medical genetics ➔ Perceived benefit of nutrigenomics	0.192	4.475***	Supported
Trust in key players ➔ Perceived benefit of nutrigenomics	0.348	6.505***	Supported
Engagement with medical genetics ➔ Perceived risk of nutrigenomics	−0.140	2.664**	Supported

*P < 0.05; **P < 0.01; ***P < 0.001

### Structural relationships

**Fig. 2.**
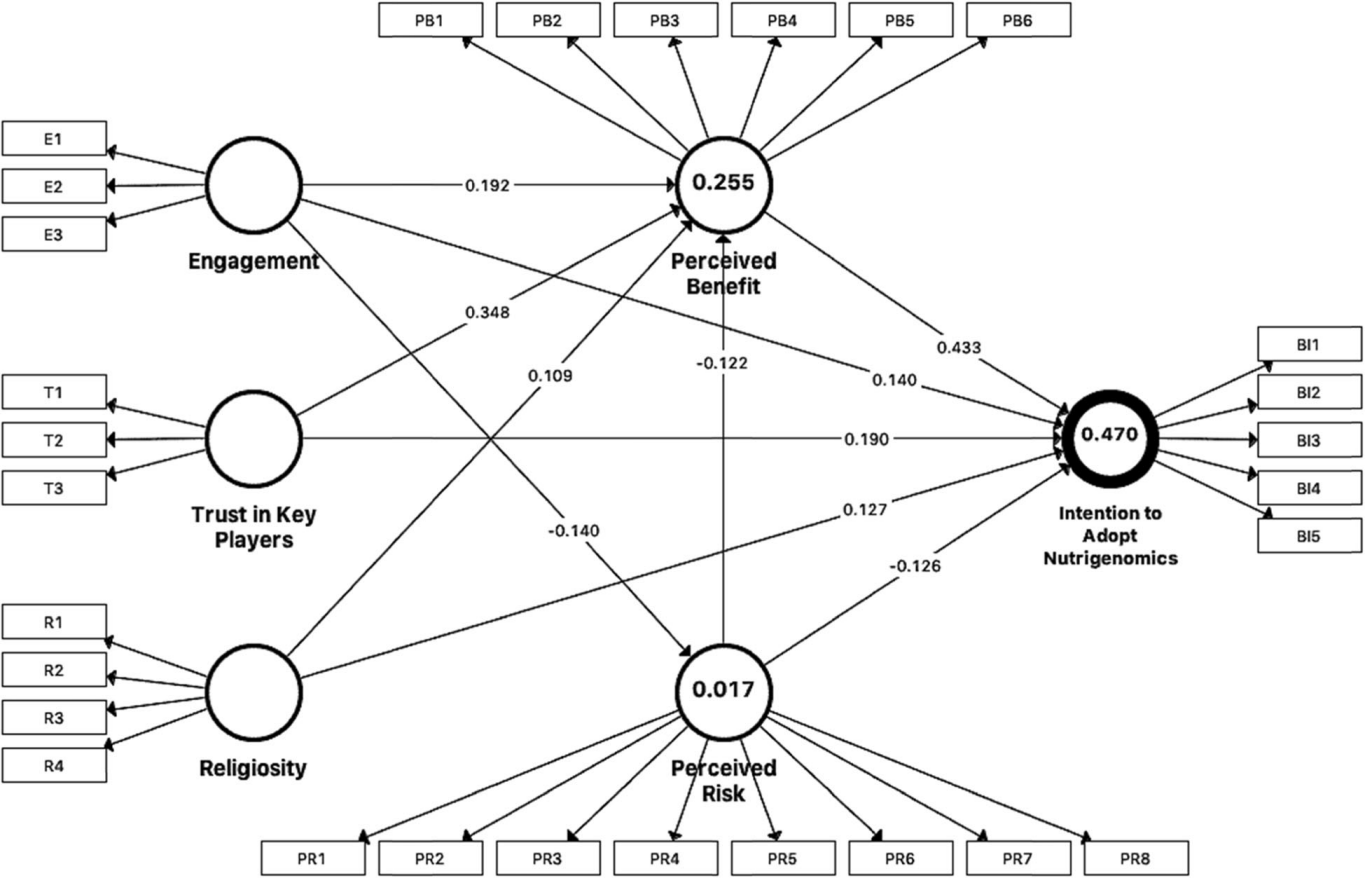
Structural equation model of factors predicting stakeholders’ intention to adopt nutrigenomics. The details for items E1-E3, T1-T3, R1-R4, PB1-PB6, PRI1-PRI8, and BI1-BI3 are presented in Table 2

A PLS-SEM algorithm was used to assess the size of the path coefficients, whilst the significance of the relationships amongst the variables was tested using a bootstrapping procedure with 5000 resamples. The path coefficient (*ß*) and the squared multiple correlations (*R*^2^) were used to measure the explanatory power of the model. The *R*^2^ of the endogenous latent variable (intention to adopt nutrigenomics) was 0.47, which indicates that the model has substantial explanatory power [110], explaining 47% of the variance in intentions to adopt nutrigenomics (Fig. 2).

As Fig. 2 and Table 6 show, the perceived benefits are the main direct predictor of the intention to adopt nutrigenomics (*ß* = 0.433, *P* < 0.001), followed by trust in key players (*ß* = 0.190, *P* < 0.001), engagement with medical genetics (*ß* = 0.140, *P* < 0.001), religiosity (*ß* = 0.127, *P* < 0.01) and perceived risks (*ß* = −0.126, *P* < 0.001). Perceived benefits were also a mediator for the relationship between the four factors and the intention to adopt nutrigenomics. The factors significantly associated with perceived benefits were perceived risks (*ß* = −0.122, *P* < 0.01), trust in key players (*ß* = 0.348, *P* < 0.001), engagement with medical genetics (*ß* = 0.192, *P* < 0.001) and religiosity (*ß* = 0.109, *P* < 0.005). Perceived risks also mediated the relationship between engagement with medical genetics and intention to adopt nutrigenomics (*ß* = −0.140, *P* < 0.01). The results of hypotheses testing for the structural model are presented in Table 6.

## Discussion

The results show that perceived benefits were the most important predictor of an individual’s intention to adopt nutrigenomics, whilst perceived risks were only weakly related to intention. Although the Malaysian stakeholders appear to have assessed both the benefits and risks of nutrigenomics, these results suggest that during the decision-making process, they tended to focus more on the beneficial aspects. This means that, when the respondents perceive the technology as having more benefits than risks, their decision is based on the stronger of the two perceptions. It is well-established, from various studies that perceived benefits have a strong relationship with both intention to adopt nutrigenomics-based personalised nutrition, and positive attitude towards nutrigenomics-based personalised nutrition, at least in the European cultural context [29, 32, 111, 112]. A qualitative study using focus groups involving 124 respondents was also conducted in eight European countries—the UK, Spain, the Netherlands, Poland, Portugal, Ireland, Greece and Germany—which summarised the findings in terms of nutrigenomics-based personalised nutrition appearing to construe potential benefits to individual and public health, which translates into an important factor in terms of their uptake [25]. Other studies also suggested that many European and US citizens positively support the use of genetic testing, as they were of the opinion that such tests would bring benefits with regard to heritable diseases [65, 113]. However, high risk perceptions associated with the development of the tests may negatively influence attitudes towards genetic testing [114, 115]. The perceived benefit-risk balance is important when people make decision whether to support nutrigenomics. Some scientists anticipate the existence of an inverse relationship between these two factors, which has an association with technological acceptance [37, 111]. Furthermore, studies by Poínhos et al. [111] and Frewer [116] showed that people who perceive nutrigenomics-based personalised nutrition as offering specific benefits tend to perceive it having fewer risks and express a greater behavioural intention to adopt. It would be interesting to investigate whether an inverse relationship exists between perceived benefits and perceived risks, which could not be determined in this model due to the limitations of the PLS-SEM software. In the future, it is suggested that this association be determined using other software that can test non-recursive relationships such as CB-SEM.

Other factors such as trust in key players, engagement with medical genetics and religiosity also significantly influence stakeholders’ intentions regarding the adoption of nutrigenomics. Trust in key players was found to have a significant positive association with intention. This suggests that when respondents have high trust in the key players who are responsible for regulating the technology, such as service providers, industry figures and government regulators, they are more likely to intend to adopt the technology. This is in line with previous findings showing that the trust factor represents a significant and relevant predictor associated with consumers’ intention to adopt nutrigenomics-based personalised nutrition [70] and the acceptance of different novel food technologies [117–119]. Along the same lines, trust is a significant contributory factor to people’s risk and benefit assessment of gene technology [54, 120, 121]. Without trust in key players, the public will have greater perceptions of the potential technological risks, and will be more sceptical about the assurances provided by experts and regulators [65, 122].

Engagement with medical genetics was found to be positively related to the intention to adopt nutrigenomics. People who are more exposed to the issues surrounding medical genetics are expected to be more supportive of such technology. Asadollahi et al. [123] suggested that greater public awareness and knowledge with regard to medical genetics and its services is required to facilitate the implementation and establishment of genetic services, which may lead to the successful development of future medical contexts. In line with this, a qualitative study by Hann et al. [124] suggested that low levels of both awareness and knowledge were highlighted as barriers to the adoption of genetic testing and genetic counselling. Notably, better public education and genetic literacy especially in low and middle-income countries may improve understanding and, consequently, increase support for the application of genetic services [125]. However, the influence of knowledge on attitudes towards science and technology [71, 72] and attitudes towards nutrigenomics and genetic testing [29, 31, 86, 126–128] was found to be inconsistent. On the other hand, the significant role of the engagement factor in predicting support towards various biotechnology applications was emphasised by Gaskell et al. [65], whilst the influence of involvement on the positive affect and benefits of nutrigenomics was reported by Pin [35].

It is interesting to note that in this study, religiosity was found to have a positive association with the intention to adopt nutrigenomics and to have a significant positive association with the perceived benefits; this indicates that respondents who claim to have a high level of religiosity also see the benefits of nutrigenomics. Previous studies reveal mixed findings regarding the influence of religiosity on attitudes to science and technology. Several studies have reported a positive relationship between being religious and having a positive attitude towards science [88] and genetic engineering [87, 129]. The role of religiosity in shaping an individual’s preferences has been discussed in previous studies [130, 131]; however, there has been no research on their possible role in relation to nutrigenomics, including within Muslim majority communities. Evidence from past research suggests that this factor may affect health-promoting behaviour in terms of risk reduction [132]. Evidence from a number of experimental studies has established that the health benefits associated with the consumption of functional food is the main reason for their acceptance [133–135]. The Malaysian stakeholders in this study perceived the ability of nutrigenomics to bring about high benefits to their health, thus having a high intention to adopt the technology. The influence of cultural values on people’s attitudes and their behaviour, with regard to food choice decisions and eating habits, have been documented [77, 78]. According to Hassan [78], religion is an ongoing part of the daily life of Muslims and is embedded in their cultural and personal values. Functional foods have been associated with various cultures and ethnic groups for centuries in Malaysia [77]. The major respondents in this study were Malays: the dominant ethnic group representing more than half of the nation’s population (63.1%) [83]. Malay Muslim individuals consume functional foods to achieve certain standards in their lives in terms of their cultural and personal values, which also reflect Islamic values. The *Quran* and *Sunnah* outline the teachings that show every Muslim how to protect their health and live their life in a state of purity [78], and food plays a vital role in the health and daily life of a believer. In addition, Islam also encourages the use of science and technology to improve human life as long as the application brings benefits (*maslahah*) and minimises harm (*mafsadah*) to society and the environment [136]. This may also explain the reason for the high level of support relating to the intention to adopt nutrigenomics in a developing multicultural country such as Malaysia.

It is also pertinent to note that perceived benefits are mediators for all other predicting factors, which emphasises its prominent role in the model. Benefits to patients such as better health outcomes and improved quality of life can be achieved with nutrigenomics. In addition, nutrigenomics can help health professionals improve health recommendations and solve issues linked to specific dietary compounds [137]. Perceived risks have a significant negative association with perceived benefits, suggesting that people who see nutrigenomics as less risky will generally see its potential benefits as being high. Trust in key players was seen to positively influence the perceived benefits. Previous studies by Chen and Li [43], Costa-Font and Gil [47], and Amin et al. [138] also found a positive association of a similar strength between the trust in key players and perceived benefits. This demonstrates that when the respondents’ perceptions of key players such as service providers, industry figures and government regulators are high, they perceive nutrigenomics as being more beneficial. This then translates into higher levels of support for adopting the technology. This finding is similar to that of Pin [35], who reported that the trust factor significantly contributed to indirect predictors regarding the adoption of nutrigenomics. Engagement with medical genetics was positively related to the perceived benefits of nutrigenomics. Stakeholders who were highly engaged with medical genetics tended to perceive nutrigenomics as being more beneficial, whilst at the same time perceiving there were lower risks associated with the application. This demonstrates the significance of the engagement with the medical genetics factor in affecting the positive acceptance of nutrigenomics. Pin [35] stated that engagement is a predictor for both the cognitive process (the perceptions of benefits and costs) and the affective process (positive and negative affective evaluation), which in turn influences people’s intention to adopt new technologies.

The aforementioned predictors were directly related to the intention to adopt nutrigenomics and were associated with the intentions through the perceived benefits. This supports the crucial role of perceived benefits in predicting intentions to adopt nutrigenomics. It is important that researchers and practitioners recognise perceived benefits as a key indicator of intentions to adopt. Therefore, to enhance public acceptance, more effective communication is required to raise awareness about the promises offered by new technologies such as nutrigenomics. Perceived risk also serves as a mediator for the relationship between engagement with medical genetics and intention to adopt. Engagement with medical genetics negatively influences the perceived risk, which indicates that when people are less engaged with medical genetics, they perceive more risks and are less eager to adopt nutrigenomics, which supports the previous findings. It is important to note that items relating to perceived moral concerns were grouped together with perceived risks, so that moral concerns were not conceptualised as a separate construct. Frewer [116] found that, in the case of nutrigenomics-based personalised nutrition, perceived barriers were linked to social structures and practices rather than ethical concerns, although the cultural context of the research was European.

It is, however, important to recognise several limitations of the study dealt with in this paper. The data from both stakeholders were combined, as both stakeholders were expected to be the main potential beneficiaries of nutrigenomics with the same interests, as well as by looking at their descriptive responses, whereby their pattern of responses was found to be similar. The combined model will be useful to provide an initial understanding of the causal relationship between the determinants of the Malaysian stakeholders’ intentions to adopt nutrigenomics. However, generalising the model beyond this population is not recommended. Additional measures have been carried out in the study to select only good indicators based on the validity and reliability of the measurements. In future, it will be good to carry out multi group analyses and compare the findings with this study. It is also recommended that this model be cross-validated to ascertain whether or not these results are valid and can be generalised across other stakeholder groups and regions. Another shortcoming is that the predictive factors used in this study to assess the intention to adopt nutrigenomics are not exhaustive. However, the model in this study helps in identifying the predictors that can serve as a useful evidence baseline for scientists, governments and policy makers for further development of nutrigenomics and the possible innovations emerging from it. Future research should consider other factors such as those related to service attributes and privacy issues [25, 32] that may influence consumers’ acceptance of nutrigenomics, and also involve a wider diversity of stakeholders such as the general public, business people, policy makers and consumer interest groups.

## Conclusion

The findings of this study suggest that the intentions of Malaysian stakeholders to adopt nutrigenomics is characterised by a complex decision-making process involving interrelated factors. The perceived benefit-perceived risk balance is a crucial element in deciding whether or not to support nutrigenomics. When stakeholders perceive that the benefits of nutrigenomics exceed the risks, the perceived benefits are significant in influencing and predicting their intention to adopt nutrigenomics. The crucial role of perceived benefits as a direct predictor and mediator for all other predictors should be noted by researchers and practitioners. Therefore, it is expected that increased exposure to the beneficial aspects of nutrigenomics, as well as to the processes involved, would influence the beneficial perceptions and thus support the application. The influence of other factors, for example, trust in key players and religiosity should also be considered when devising an appropriate technology acceptance strategy. The model developed identifies important predictors of Malaysian stakeholders’ intention to adopt nutrigenomics.

## Supplementary information

**Additional file 1.** NUTRIGENOMICS

**Additional file 2.** ITEMS FOR MEASUREMENT
