# Supplementary material for: Predictors of stakeholders’ intention to adopt nutrigenomics
Source: Genes Nutr. 2020 Sep 22;15:16. doi: 10.1186/s12263-020-00676-y (PMC7509940; doi:10.1186/s12263-020-00676-y)
Supplement: Supplementary file 1 — Additional file 1. NUTRIGENOMICS [file 12263_2020_676_MOESM1_ESM.docx]

**APPENDIX 1**

**NUTRIGENOMICS**


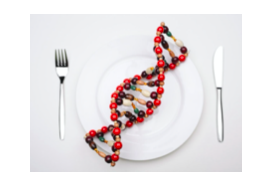


**DEFINITION**

The study of how foods affect our genes and how individual genetic variations can affect how we respond to nutrients in the foods we eat, both of which may impact the risk of developing nutrition-related chronic diseases, such as heart disease, diabetes, and certain cancers. (<https://definitionsonline.com/wellness-facility/nutrition/>)


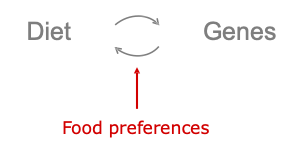


**GOALS**

1. To identify genes and gene variants that may be significant in understanding genetic responses to diet.
2. To identify genotypes associated with diet-related disease.
3. To modify diet for treatment or prevention of disease.
4. To improve dietary guidelines for group and individual levels.

**APPLICATIONS OF NUTRIGENOMICS**

Obesity

1. Obesity is one of the most widely studied topics in nutrigenomics.
2. Multiple studies have found association between SNPs (pronounced “snips”) and obesity. SNPs are the most common type of genetic variation among people.
3. Most well-known obesity associating gene is the FTO gene. Certain variants of the gene appear to be correlated with obesity in humans.
4. Among studied individuals, it was found that those with AA genotype showed a higher BMI compared to those with TT genotype when having high fat or low carbohydrate dietary intake.

Example: Caffeine

1. Researchers have shown that people who has slow caffeine metabolism are much more susceptible to complication associated with high blood pressure as well as increased risk of heart attack.
2. Therefore, slow caffeine metabolizers should not drink more than one cup of coffee a day.


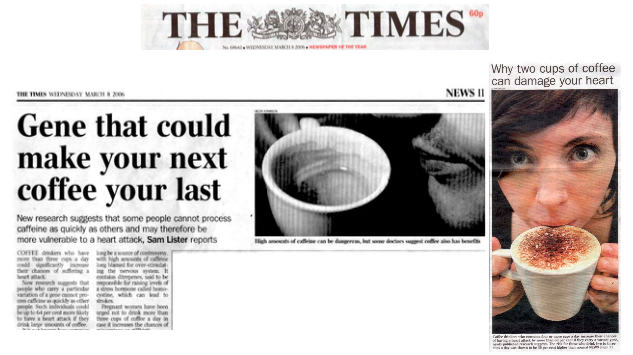


**ISSUES & LIMITATIONS**

1. Because of the complex nature of foods, including nutrient variability, nutrient-nutrient interactions, and the genetic variations among individuals who consume a certain diet, it will be difficult to determine cause and effect with certainty.
2. The field of nutrigenomics has the potential to generate overwhelming quantities of data. How to classify and interpret this data will inevitably present unique challenges.
