# Supplementary material for: Predictors of stakeholders’ intention to adopt nutrigenomics
Source: Genes Nutr. 2020 Sep 22;15:16. doi: 10.1186/s12263-020-00676-y (PMC7509940; doi:10.1186/s12263-020-00676-y)
Supplement: Supplementary file 2 — Additional file 2. ITEMS FOR MEASUREMENT [file 12263_2020_676_MOESM2_ESM.docx]

**APPENDIX 2. ITEMS FOR MEASUREMENT**

**Engagement**

1. **Past and intended information seeking behaviour (item 1-5)**

To what extent, do you agree with the following statements about medical genetics?

|  | **Strongly Strongly**  **disagree agree** |
| --- | --- |
| 1. Before this survey, I often talked about medical genetics with other people. | 1 2 3 4 5 6 7 |
| 1. I often involved in public discussions or hearings about medical genetics. | 1 2 3 4 5 6 7 |
| 1. I often take the time to read articles from newspapers or magazines, or watch television program on medical genetics. | 1 2 3 4 5 6 7 |
| 1. I often read about medical genetics from the internet. | 1 2 3 4 5 6 7 |
| 1. I often participate in online discussions about medical genetics through online chatting, forum, social networks, blog, etc. | 1 2 3 4 5 6 7 |

1. **Awareness (item 6-12)**

Have you heard about the following things before this survey?

|  | **Yes** | **No** |
| --- | --- | --- |
| 1. Genetic tests that analyses your DNA to identify potential health risks | 1 | 2 |
| 1. Human genome project to map human genes | 1 | 2 |
| 1. DNA testing company in Malaysia such as EasyDNA, The LifeStyle Clinic & DNA Profiling Malaysia. | 1 | 2 |
| 1. Gene Therapy: a technique that uses genes to treat or prevent disease. | 1 | 2 |
| 1. Pharmacogenomics: study how genes affect a person’s response to drugs. | 1 | 2 |
| 1. Nutrigenomics: study of the interaction of foods and genes, with the goal of using diet to prevent or treat disease. | 1 | 2 |
| 1. Guidelines on Ethical Issues in Medical Genetics and Genetic Service in Malaysia. | 1 | 2 |

1. **Knowledge (item 13-22)**

For each of the following statements, please indicate whether you think it is true or false.

|  | **True** | **False** | **Don’t know** |
| --- | --- | --- | --- |
| 1. Identical twins have the same genes. | 1 | 2 | 3 |
| 1. DNA is genetic material that stores heritable information. | 1 | 2 | 3 |
| 1. Half of your genes come from your mother and half from your father. | 1 | 2 | 3 |
| 1. If a person is the carrier of a disease gene it means that he/she has the disease. | 1 | 2 | 3 |
| 1. A normal person carries 46 chromosomes in their cells. | 1 | 2 | 3 |
| 1. Your white blood cells contain a full copy of all your genes. | 1 | 2 | 3 |
| 1. It is the father’s chromosome that decide if a baby is a boy or girl. | 1 | 2 | 3 |
| 1. Healthy parents can have a child with an inherited disease. | 1 | 2 | 3 |
| 1. Each human body is estimated to contain as many as 23,000 genes. | 1 | 2 | 3 |
| 1. The Chimpanzee is the animal species genetically closest (99%) to mankind. | 1 | 2 | 3 |

**Trust in key players (item 23-25)**

To what extent that the following institutions/group have done a good job for the society?

| **Institution/group** | **Strongly Strongly**  **disagree agree** |
| --- | --- |
| 1. Medical doctors / Medical geneticist / Pharmacologist / Nutritionist | 1 2 3 4 5 6 7 |
| 1. Govt. dept. involved in medical genetics regulation such as Ministry of Health. | 1 2 3 4 5 6 7 |
| 1. Genetic Testing Companies / Pharmaceutical / Nutraceutical Industries | 1 2 3 4 5 6 7 |

**Religiosity (item 26-29)**

Please circle your level of agreement with the following statements:

| Items | **Strongly Strongly**  **disagree agree** |
| --- | --- |
| 1. Religion is important in my life | 1 2 3 4 5 6 7 |
| 1. Religious views are important when I have to make decisions about controversial issues | 1 2 3 4 5 6 7 |
| 1. Praying is important in my life | 1 2 3 4 5 6 7 |
| 1. Reading scriptures is important in my life | 1 2 3 4 5 6 7 |

**Perceived Benefit of Nutrigenomics (item 30-35)**

**Nutrigenomics:** study of the interaction of foods and genes, with the goal of using diet to prevent or treat disease.

Please circle the number that corresponds to your answer for each question.

|  | **Strongly Strongly**  **disagree agree** |
| --- | --- |
| 1. Nutrigenomics will make people become healthier | 1 2 3 4 5 6 7 |
| 1. How much do you agree that nutrigenomics will bring cure for chronic diseases? | 1 2 3 4 5 6 7 |
| 1. Nutrigenomics will enhance the quality of life of the Malaysian society | 1 2 3 4 5 6 7 |
| 1. Nutrigenomics will bring benefits to the future generations | 1 2 3 4 5 6 7 |
| 1. The benefits of nutrigenomics will be greater than the harm it may cause | 1 2 3 4 5 6 7 |
| 1. Nutrigenomics enable the society to solve problems that currently cannot be solved by the conventional method. | 1 2 3 4 5 6 7 |

**Perceived Risk of Nutrigenomics (item 36-43)**

**Nutrigenomics:** study of the interaction of foods and genes, with the goal of using diet to prevent or treat disease.

Please circle the number that corresponds to your answer for each question.

|  | **Strongly Strongly**  **disagree agree** |
| --- | --- |
| 1. How worried are you about using nutrigenomics that may give risk to unknown consequences? | 1 2 3 4 5 6 7 |
| 1. Nutrigenomics will do more harm than good for society. | 1 2 3 4 5 6 7 |
| 1. To what extent do nutrigenomics will lead to any danger to the Malaysian society? | 1 2 3 4 5 6 7 |
| 1. Any harmful effect using nutrigenomics will only manifest itself after long term duration. | 1 2 3 4 5 6 7 |
| 1. I am worried about the safety of nutrigenomics. | 1 2 3 4 5 6 7 |
| 1. Nutrigenomics is threatening the natural order of things. | 1 2 3 4 5 6 7 |
| 1. Nutrigenomics may give rise to ethical issues. | 1 2 3 4 5 6 7 |
| 1. Nutrigenomics is like “Playing God”^.^ | 1 2 3 4 5 6 7 |

**Intention to Adopt Nutrigenomics (item 44-48)**

**Nutrigenomics**: study of the interaction of foods and genes, with the goal of using diet to prevent or treat disease.

Please circle the number that corresponds to your answer for each question.

|  | **Strongly Strongly**  **disagree agree** |
| --- | --- |
| 1. I am willing to take nutrigenomics if it can cure my disease | 1 2 3 4 5 6 7 |
| 1. I am willing to support nutrigenomics. | 1 2 3 4 5 6 7 |
| 1. I am willing to recommend nutrigenomics to my family or close friends | 1 2 3 4 5 6 7 |
| 1. If I am able to pay for nutrigenomics, I would not hesitate to take it. | 1 2 3 4 5 6 7 |
| 1. If nutrigenomics is cheaper than existing treatment, I would not hesitate to take it. | 1 2 3 4 5 6 7 |
